# Prognostic significance of circulating tumor DNA detection and quantification in cervical cancer: a systematic review and meta-analysis

**DOI:** 10.3389/fonc.2025.1566750

**Published:** 2025-04-04

**Authors:** Xiumin Zhao, Shufu Hou, Ruiqi Hao, Yelei Zang, Dandan Song

**Affiliations:** ^1^ Department of Neurology, Shandong Provincial Third Hospital, Cheeloo College of Medicine, Shandong University, Jinan, China; ^2^ Department of Gastrointestinal Surgery, Central Hospital Affiliated to Shandong First Medical University, Jinan, China; ^3^ Department of Gastrointestinal Surgery, Xintai City People’s Hospital, Xintai, Shandong, China; ^4^ Department of General Surgery, The First Affiliated Hospital of Shandong First Medical University, Jinan, China

**Keywords:** cervical cancer, circulating tumor DNA, overall survival, progression-free survival, disease free survival

## Abstract

**Background:**

Circulating tumor DNA (ctDNA) is an emerging biomarker in cervical cancer, with elevated levels typically indicating a higher tumor burden. However, its prognostic value in cervical cancer patients remains debated. This meta-analysis aims to clarify the prognostic significance of ctDNA in this patient population.

**Methods:**

We searched the PubMed, Cochrane Library, CNKI, and EMBASE databases for studies published up to September 30, 2024, to investigate the prognostic significance of ctDNA in cervical cancer patients. The outcome measures included overall survival (OS) and progression-free survival (PFS)/disease-free survival (DFS).

**Results:**

This analysis included 10 studies encompassing a total of 706 cervical cancer patients. Findings revealed that patients with detectable baseline ctDNA had significantly poorer OS(HR = 1.64, 95% CI = 1.45–1.86, P < 0.001) as well as worse PFS or DFS (HR = 1.42, 95% CI = 1.07–1.89, P = 0.015). Additionally, ctDNA detectability during treatment was strongly associated with poorer OS (HR = 17.22, 95% CI = 4.43–66.89, P < 0.001) and PFS/DFS (HR = 4.16, 95% CI = 2.57–6.73, P < 0.001).

**Conclusions:**

This meta-analysis demonstrates that elevated ctDNA levels are significantly associated with poorer PFS, DFS, and OS in patients with cervical cancer. However, data regarding the association between ctDNA levels and OS are relatively limited, and the number of included studies remains small, with a potential risk of publication bias. Based on the current evidence, ctDNA shows promise as a valuable tool for pre-treatment assessment and an effective biomarker for monitoring therapeutic response and disease progression. Further large-scale, prospective studies are warranted to validate these findings and establish their reliability and clinical applicability.

**Systematic Review Registration:**

inplasy.com, identifier INPLASY2024120083.

## Introduction

Cervical cancer (CA) represents one of the major health challenges for women worldwide, ranking as the fourth most common malignancy among females (1, 2). Over recent years, the age of onset has been steadily decreasing, particularly for cervical adenocarcinoma. Although the exact etiology of cervical cancer remains unclear, numerous risk factors have been identified, including the number of sexual partners, parity, high-risk human papillomavirus (HPV) infection, and compromised immune function (3–5).Cervical cancer often progresses asymptomatically, with over half of the cases lacking clinical manifestations and being detected incidentally during routine gynecological examinations. Consequently, many patients are diagnosed at advanced stages (6, 7). Existing screening methods, such as the Pap smear and colposcopy, face limitations due to subjective interpretation, resulting in high rates of false negatives and positives (8–10).With the advent of “precision medicine” and advancements in molecular biology and sequencing technologies, ctDNA has garnered significant attention as a non-invasive and safe tool for the diagnosis of malignant tumors (11). Next-generation sequencing (NGS) has further improved the accuracy and efficiency of ctDNA detection, highlighting its potential role in early cancer diagnosis, treatment monitoring, and relapse prediction across various malignancies. Consequently, ctDNA has emerged as a promising molecular biomarker (12–18).

ctDNA refers to fragments of free DNA released into the bloodstream by tumor cells, carrying tumor-specific genetic alterations such as mutations, rearrangements, and copy number variations (19, 20). Reflecting real-time tumor dynamics within approximately a week, ctDNA enables clinicians to monitor disease progression dynamically (21). Its non-invasive nature allows extraction from blood or other body fluids, eliminating the need for traditional tissue biopsies and establishing it as a cornerstone of “liquid biopsy” technology. Early studies, such as those by Vasioukhin et al. (22), utilized PCR to detect RAS proto-oncogene mutations in leukemia patients’ plasma. Subsequent research has identified tumor-related genetic alterations in plasma ctDNA across multiple malignancies (23). In breast cancer, Mariko et al. (16) demonstrated the tumor-specific frequent methylation of the HOPX gene, which was associated with invasive potential, positioning it as a biomarker for treatment monitoring. Similarly, HungChih et al. (17) reported that mutations in KRAS, BRAF, and NRAS were correlated with poor progression-free survival in metastatic colorectal cancer patients. In ovarian cancer, Boyd et al. (24) found that FBXW7 mutations were linked to chemoresistance, offering new therapeutic and diagnostic opportunities. Although extensive studies have established the clinical value of ctDNA in various malignancies, research on its application in cervical cancer remains limited. To address this gap, we conducted a systematic meta-analysis of published studies to evaluate the prognostic value of ctDNA in cervical cancer patients, aiming to provide robust evidence for its future clinical applications.

## 2 Materials and methods

### 2.1 Search strategy

This systematic review and meta-analysis were conducted in strict adherence to the guidelines set forth in the Preferred Reporting Items for Systematic Reviews and Meta-Analyses (PRISMA) (25), with the aim of evaluating the prognostic significance of ctDNA in melanoma patients receiving immune checkpoint inhibitor therapy. To ensure the comprehensiveness and scientific rigor of the findings, the research team consisted of two independent researchers who systematically searched four major databases: PubMed, Embase, CNKI (China National Knowledge Infrastructure), and the Cochrane Library to identify relevant studies. The search covered all studies published from the inception of these databases until September 30, 2024.The literature search was performed using the following keywords:”Uterine Cervical Neoplasm” or “Cervix Neoplasm” or “Cervical Neoplasm” or “Cancer of the Uterine Cervix”or “Cancer of the Cervix” or “Cervix Cancer” or “Uterine Cervical Cancers” or”Uterine Cervical Cancer” and “ctDNA” or “circulating tumor DNA”. These terms were chosen to ensure a thorough retrieval of studies related to ctDNA in melanoma patients treated with immune checkpoint inhibitors. Additionally, both free-text search terms and Medical Subject Headings (MeSH) were utilized to search within titles and abstracts, further enhancing the comprehensiveness of the search. To further augment the thoroughness of our literature review, we also screened the references of all included studies to ensure that no potentially relevant high-quality studies were overlooked.

### 2.2 Inclusion and exclusion criteria

#### 2.2.1 Inclusion criteria

(1) Cervical cancer patients confirmed by gold standard pathological diagnosis; (2) Clinical studies related to the prognostic value of circulating tumor DNA; (3) Studies providing direct or indirect outcomes related to overall survival and progression-free survival in cervical cancer patients, including but not limited to hazard ratios and 95% confidence intervals (CI).

#### 2.2.2 Exclusion criteria

(1) Studies that include only cell-free DNA (cfDNA) data without relevant data on cervical cancer patients; (2) Case reports, conference abstracts, animal studies, or review articles; (3) Studies lacking sufficient and valid data to estimate HR and 95% CI; (4) Duplicate publications of data.

### 2.3 Data extraction and quality assessment

Data extraction was carried out by two independent researchers, and any discrepancies were resolved through discussion or consultation with a third researcher. The extracted data included the first author’s name, year of publication, study location, study design, sample size, mean or median patient age, cancer stage, treatment methods, detection techniques, timing of sample collection, target genes, median follow-up period (in months), and survival analysis outcomes (including hazard ratios (HR) and 95% confidence intervals (CI) for OS and PFS/DFS. Study quality was assessed using the Newcastle-Ottawa Scale (NOS), which evaluates three key domains: selection (0–4 points), comparability (0–2 points), and outcome assessment (0–3 points). Each researcher independently scored the eight questions within these domains, with a total possible score ranging from 0 to 9. Studies scoring over 6 points were classified as high quality (26).

### 2.4 Statistical methods

This study utilized Stata SE (version 16.0; StataCorp, College Station, Texas, USA) for statistical analysis to investigate the potential association between ctDNA and OS as well as PFS/DFS. Hazard ratios (HR) with 95% confidence intervals (CI) were calculated under two specific conditions: (a) baseline ctDNA levels measured prior to surgery or any initial treatment; and (b) ctDNA levels measured dynamically, either once or multiple times, after the initiation of treatment. This stratified analysis enables a more comprehensive understanding of the prognostic value of ctDNA at different stages of treatment, offering valuable insights to guide clinical management.Heterogeneity across studies was assessed using Cochran’s Q-test and I² statistics, with the choice of statistical model depending on these results. A random-effects model was employed when I² > 50% or the p-value from the Q-test was < 0.10, indicating significant heterogeneity. Conversely, a fixed-effects model was applied when heterogeneity was not significant. Publication bias was initially assessed by visual inspection of funnel plot symmetry and further confirmed using Egger’s regression analysis and Begg’s test, where a p-value < 0.05 was indicative of potential publication bias. Sensitivity analyses were also performed to evaluate the influence of individual studies on the overall results, ensuring the robustness and reliability of the findings.

## 3 Results

### 3.1 Study selection and characteristics

**Figure 1:**
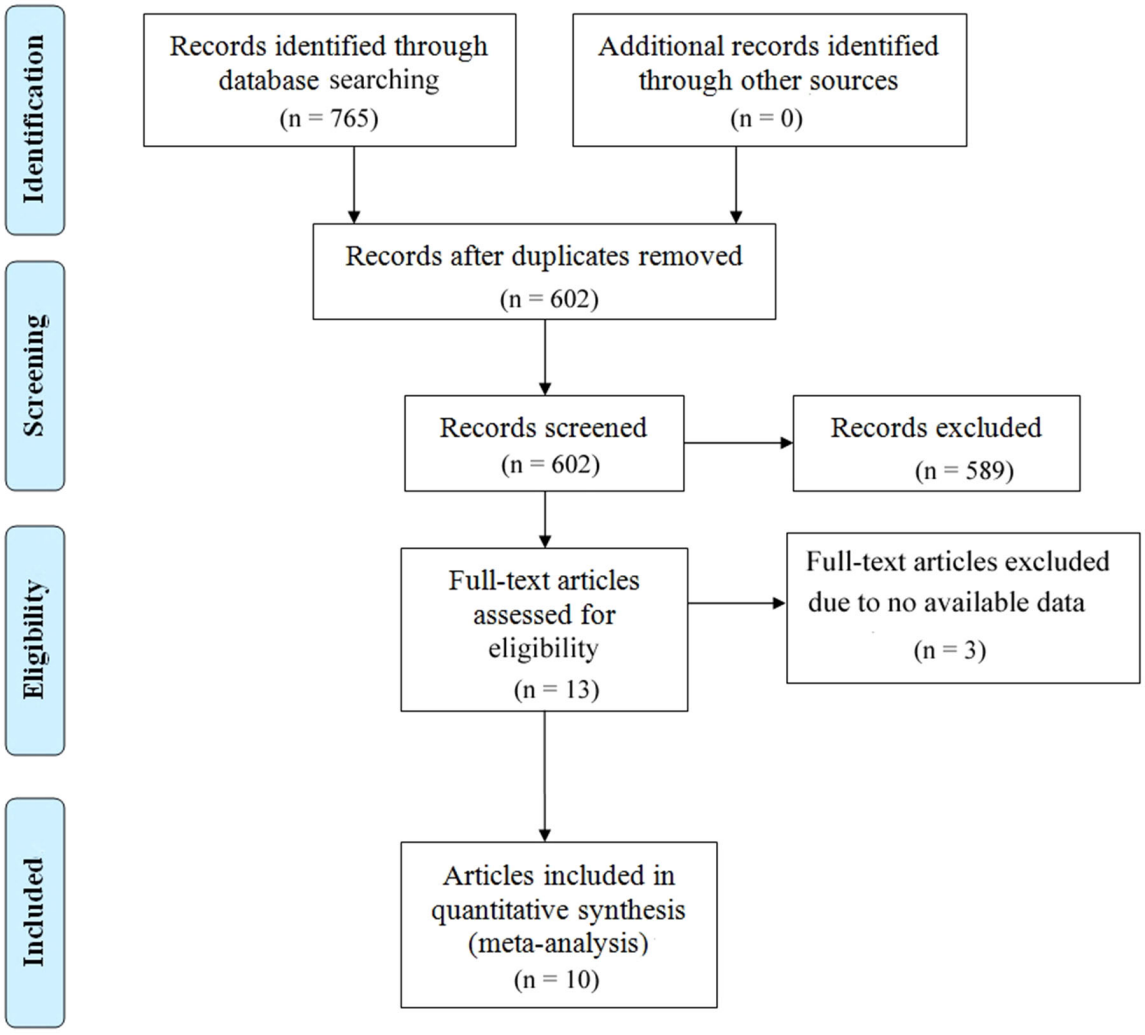
Presents the PRISMA flowchart outlining the literature selection process.

The study selection process is illustrated in **Figure 1**. A comprehensive literature search identified a total of 768 articles from multiple databases, including 217 from PubMed, 527 from Embase, 13 from CNKI, and 8 from The Cochrane Library. After removing duplicate entries, 602 unique articles remained. These were rigorously screened by evaluating their titles and abstracts against predefined inclusion and exclusion criteria. Consequently, 589 articles were excluded for failing to meet the eligibility criteria, and 3 articles were eliminated due to the lack of full-text access. Ultimately, 10 studies met the inclusion criteria and were included in the final analysis (27–36). The characteristics of the included studies are summarized in **Table 1**. These studies, published between 2018 and 2024, originated from various countries, including three from Canada, three from France, two from China, and two from Sweden. Sample sizes ranged from 18 to 188 participants, encompassing a total of 706 cervical cancer patients. Specifically, four studies investigated the association between baseline ctDNA levels and OS in cervical cancer patients, while two studies focused on OS following chemotherapy or surgical intervention. Additionally, six studies examined the relationship between baseline ctDNA levels and PFS or DFS, and seven studies analyzed PFS/DFS outcomes after chemotherapy or surgery. The quality of the included studies was assessed using the NOS, with scores ranging from 7 to 8, indicating a high standard of methodological rigor. Detailed NOS scores for each study are provided in **Table 2**.

**Table 1:** Baseline characteristics of included studies.

Study, year	Country	Duration	Sample size	Median age	Stage	Treatment	Detection methods	Target genes	Median follow-up	Time of sample collection	Survival outcome	Analysis	NOS
Han 2018 (30)	Canada	2015-2016	19	43 (31-73)	IB-IVA	Chemoradiation	dPCR	HPV-related	24 (18-31)	End of treatment	PFS	U	7
Liao 2019 (36)	China	2014-2016	188	mean:49.16	I-III	Surgery or Chemoradiation	dPCR	HPV-related	NR	Pre-treatment	DFS, OS	U, M	8
Cabel 2021 (33)	France	2011-2015	55	50 (29-78)	I-IV	Chemoradiotherapy	ddPCR	HPV-related	49.9 (10-130)	Pre-treatment/End of treatment	DFS, OS/DFS, OS	U,U/U,U	7
Jeannot 2021 (29)	France	NR	94	50 (24–80)	I-IV	Surgery or Non-surgery	ddPCR	HPV-related	16.7	Pre-treatment/End of treatment	PFS/PFS	M/M	8
Leung 2021 (34)	Canada	2014-2016	17	NR	NR	Chemoradiotherapy	dPCR	HPV-related	27.5 (4.8-48.6)	End of treatment	PFS	U	7
Tian 2021 (32)	China	2016-2018	82	56.5 (23-76)	I-IV	NR	NGS	MSG mutations	21 (6-39)	Pre-treatment	PFS, OS	U,U	7
Sivars 2022 (28)	Sweden	2019-2020	18	50.5 (35–71)	I-IV	Chemoradiotherapy	ddPCR	HPV-related	mean:24.8 (16.9-33.6)	End of treatment	PFS, OS	U,U	7
Han 2023 (31)	Canada	2017-2022	70	mean:54	IB-IVA	Chemoradiation	dPCR	HPV-related	26.4 (6-65)	End of treatment	PFS	M	8
Beaussire-Trouvay 2024 (35)	France	2010-2020	97	mean:56	IB-IVA	Chemoradiotherapy	ddPCR	HPV-related	mean:47 (2-147)	Pre-treatment	DFS, OS	U,U	7
Sivars 2024 (27)	Sweden	2019-2021	66	52 (29–84)	I-IV	Surgery and/or Non-surgery	ddPCR	HPV-related	37 (17-50)	Pre-treatment/End of treatment	PFS/PFS	U/U	7

NR, not report; overall survival; PFS, progression-free survival; ddPCR,droplet digital polymerase chain reactionmultivariate; NOS, Newcastle-Ottawa Scale.

**Table 2:** Newcastle-Ottawa Scale (NOS) for quality assessment.

Studies	Selection				Comparability	Outcome			Scores
	A	B	C	D	E	F	G	H	
Han 2018 (30)	★	★	★	★	★	★	★	–	7
Liao 2019 (36)	★	★	★	★	★★	★	–	–	8
Cabel 2021 (33)	★	★	★	★	★	★	★	–	7
Jeannot 2021 (29)	★	★	★	★	★★	★	★	–	8
Leung 2021 (34)	★	★	★	★	★	★	★	–	7
Tian 2021 (32)	★	★	★	★	★	★	★	–	7
Sivars 2022 (28)	★	★	★	★	★	★	★	–	7
Han 2023 (31)	★	★	★	★	★★	★	★	–	8
Beaussire-Trouvay 2024 (35)	★	★	★	★	★	★	★	–	7
Sivars 2024 (27)	★	★	★	★	★	★	★	–	7

A study may receive a maximum of one star for each numbered item in the Selection and Outcome categories. A maximum of two stars may be given for Comparability, as directed by the NOS. ★: It stands for one point; ★★: It stands for two points.

### 3.2 Association of ctDNA with OS and PFS

**Figure 2:**
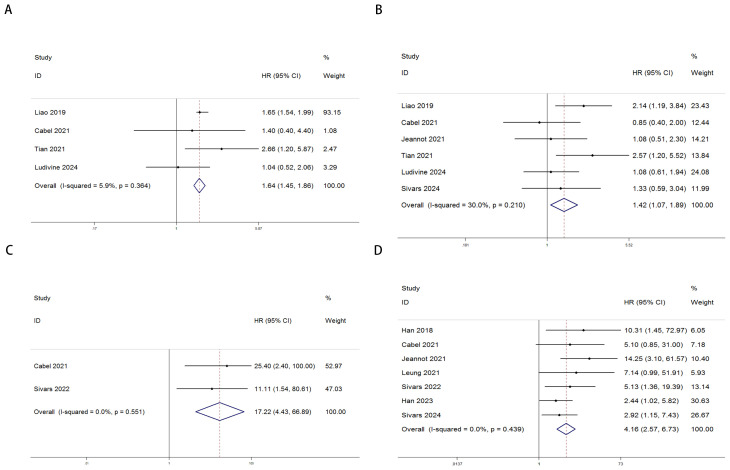
Forest plots illustrating the association between ctDNA levels and OS as well as PFS/DFS in cervical cancer patients before and after treatment [Pre-treatment: OS, **(A)**; PFS/DFS, **(B)**; Post-treatment: OS, **(C)**; PFS/DFS, **(D)**].

The relationship between ctDNA and OS, as well as PFS/DFS, in cervical cancer patients is summarized as follows: Heterogeneity testing indicated no significant heterogeneity (pre-treatment: OS: P = 0.364 > 0.1, I² = 5.9% < 50%; PFS/DFS: P = 0.210 > 0.1, I² = 30% < 50%; post-treatment: OS: P = 0.551 > 0.1, I² = 0.0% < 50%; PFS/DFS: P = 0.439 > 0.1, I² = 0.0% < 50%), suggesting that a fixed-effects model was appropriate for this meta-analysis. Independent risk estimates from four studies, along with data from six additional studies, revealed that cervical cancer patients with detectable baseline ctDNA or elevated ctDNA levels prior to ICI treatment exhibited significantly worse OS (**Figure 2A**) and PFS/DFS (**Figure 2B**) compared to patients without detectable ctDNA. The pooled hazard ratios (HRs) and 95% confidence intervals (CIs) were as follows: OS: HR = 1.64, 95% CI = 1.45–1.86, P < 0.001; PFS/DFS: HR = 1.42, 95% CI = 1.07–1.89, P < 0.001. Similarly, data from two additional studies for OS and seven studies for PFS/DFS indicated that higher post-treatment ctDNA levels were also significantly associated with poorer outcomes in terms of both OS (**Figure 2C**) and PFS/DFS (**Figure 2D**). The pooled HRs and 95% CIs were as follows: OS: HR = 17.22, 95% CI = 4.43–66.89, P < 0.001; PFS/DFS: HR = 4.16, 95% CI = 2.57–6.73, P < 0.001.Notably, due to the limited number of studies included regarding OS data for cervical cancer patients, more in-depth bias analysis and further validation have not yet been conducted. Therefore, the reliability of the above conclusions requires further investigation and confirmation. To enhance the rigor of the analysis and the credibility of the results, we focused subsequent analyses on PFS/DFS-related data, which were relatively more abundant for cervical cancer patients. Publication bias assessments, sensitivity analyses, and other validations were performed to ensure the robustness and scientific validity of the study findings.

### 3.3 Publication bias

**Figure 3:**
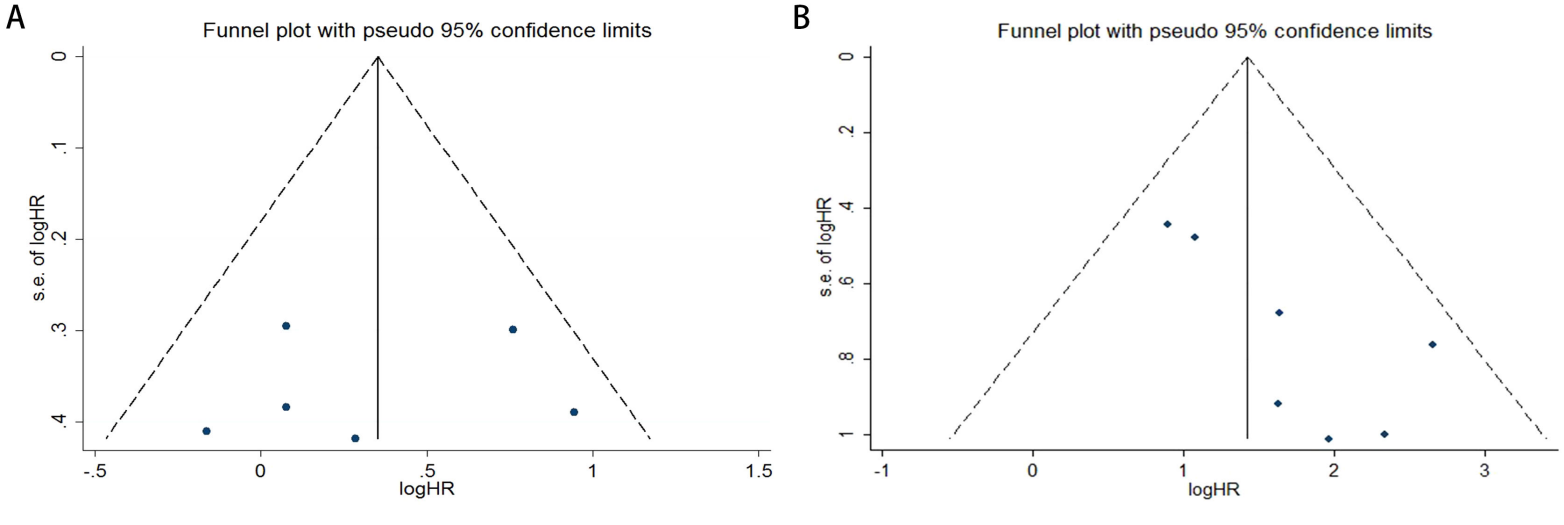
Funnel plots illustrating the assessment of publication bias in cervical cancer patients, specifically for **(A)** PFS/DFS in the pre-treatment phase and **(B)** PFS/DFS in the post-treatment phase.

**Figure 4:**
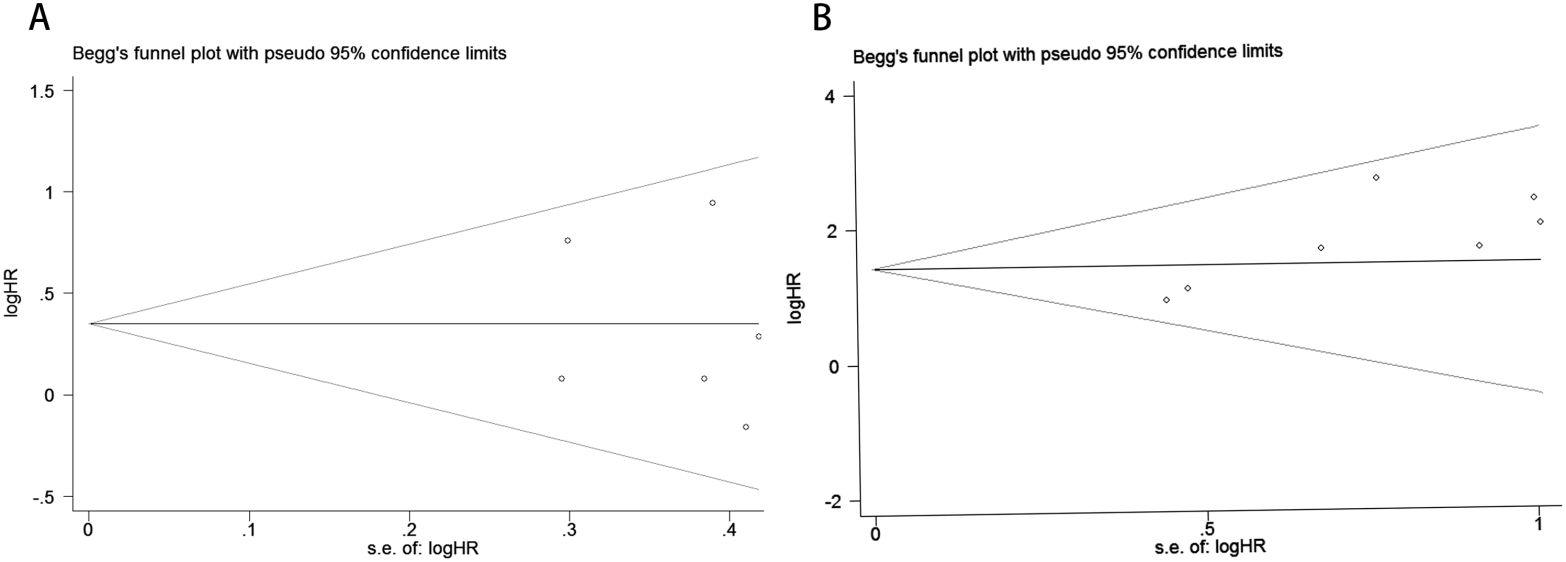
Publication bias assessment. **(A)** Begg’s test for PFS/DFS in the pre-treatment phase (p = 1.000); **(B)** Begg’s test for PFS/DFS in the post-treatment phase (p = 0.133).

**Figure 5:**
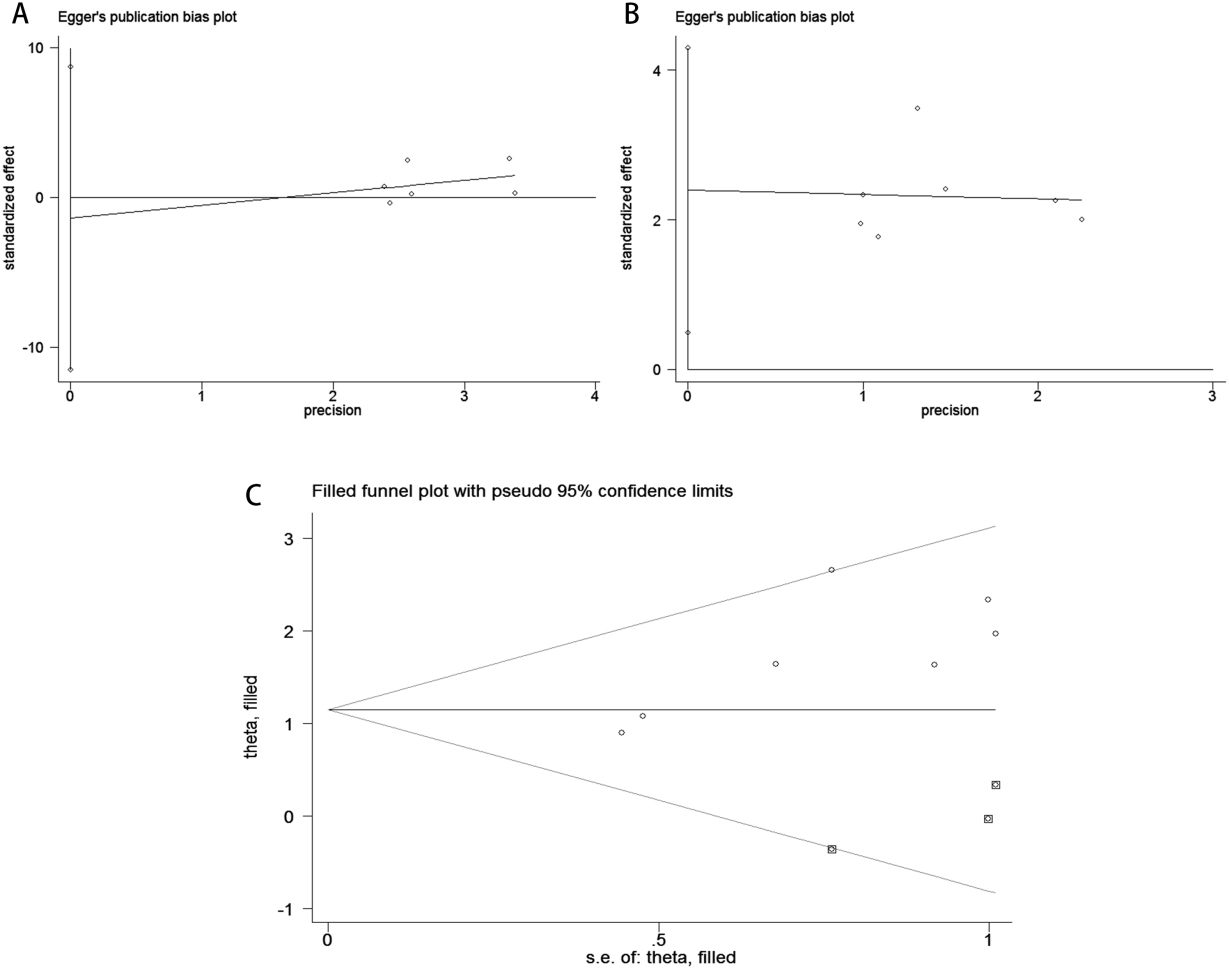
Publication bias assessment. **(A)** Egger’s test for PFS/DFS during the pre-treatment phase (p = 0.724); **(B)** Egger’s test for PFS/DFS during the post-treatment phase(p = 0.023); **(C)** Trim-and-fill adjusted Egger’s test for PFS/DFS (p = 0.158).

Publication bias was assessed using funnel plots, Egger’s linear regression analysis, and Begg’s test. The funnel plots for PFS/DFS in cervical cancer patients demonstrated favorable symmetry (**Figure 3A**: pre-treatment PFS/DFS; **Figure 3B**: post-treatment PFS/DFS). The results of Begg’s test indicated no significant publication bias for PFS/DFS before and after treatment in cervical cancer patients (pre-treatment: **Figure 4A**, p = 1.000; post-treatment: **Figure 4B**, p = 0.133). Similarly, Egger’s test showed no significant publication bias for pre-treatment PFS/DFS (**Figure 5A**, p = 0.724). However, Egger’s test results for post-treatment PFS/DFS suggested potential publication bias (**Figure 5B**, p = 0.023, P < 0.05). To further validate the robustness of the results and address potential bias, the trim-and-fill method was applied for additional analysis. After adjustment, the findings for post-treatment PFS/DFS remained statistically significant and robust, with no evidence of substantial publication bias (**Figure 5C**, p = 0.158).

### 3.4 Sensitivity analysis

**Figure 6:**
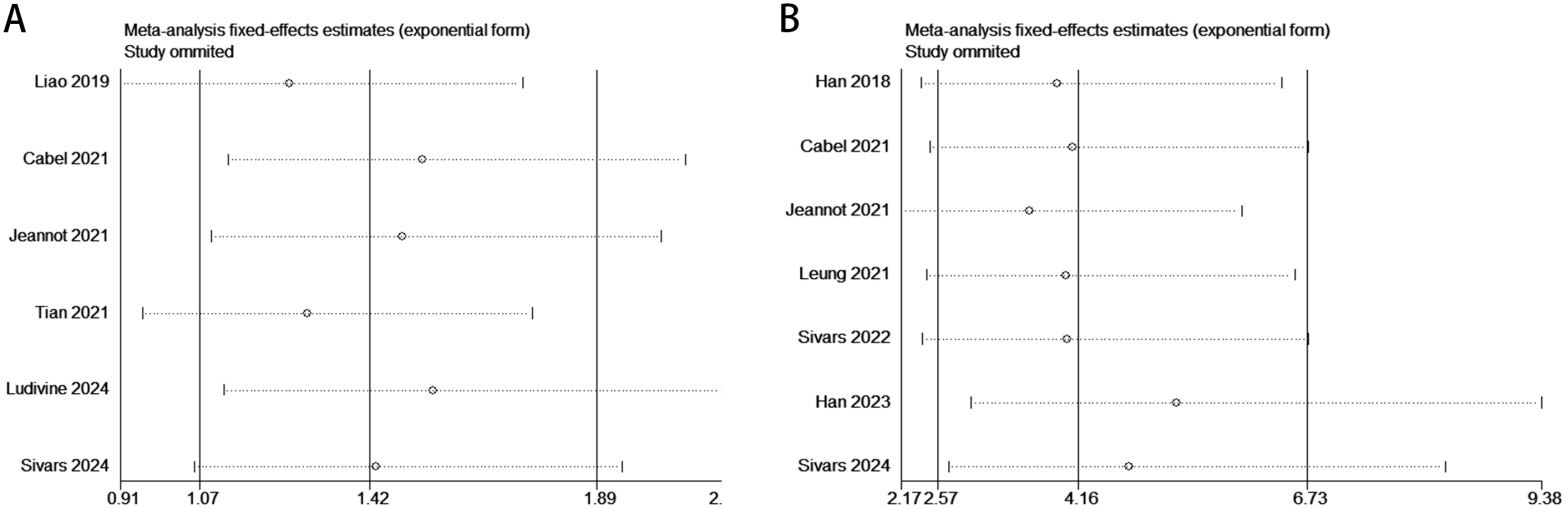
Sensitivity analysis evaluating the robustness of the pooled results between ctDNA levels and PFS/DFS in cervical cancer patients before and after treatment. [Pre-treatment: PFS/DFS: **(A)**; Post-treatment: PFS/DFS: **(B)**].

The sensitivity analysis demonstrated that the association between ctDNA and PFS/DFS in patients before and after treatment was not significantly influenced by any single study. By systematically removing each study one at a time, the analysis revealed no substantial changes in the effect size, further confirming the robustness and reliability of the results. This indicates that the overall conclusions of this study are stable and not easily affected by potential biases or outliers from individual studies, thereby enhancing the credibility of the findings (**Figure 6**).

## 4 Discussion

Cervical cancer remains a major challenge to women’s health and is the fourth most common cancer worldwide, following breast, colorectal, and lung cancers (37, 38). Among young women, cervical cancer is the second leading cause of cancer-related mortality (39). Although the progression from cervical epithelial cell hyperproliferation to malignant transformation is relatively slow, the disease is frequently diagnosed at a locally advanced stage. The prognosis of cervical cancer is the result of a complex interplay of clinical-pathological factors, HPV status, tumor microenvironment indicators, tumor markers, and other factors. While an increasing number of prognostic factors have been explored, many aspects of cervical cancer prognosis remain unclear. Tumor staging does not fully reflect the biological behavior of the tumor or the individual differences among patients; the prognostic significance of lymph node involvement is influenced by detection methods and immune microenvironment factors; while HPV status is related to the onset of cervical cancer, it cannot fully predict disease progression. Factors such as viral integration into the host cell, immune evasion, and changes in the tumor microenvironment may all influence the patient’s prognosis. Therefore, there is an urgent need for minimally invasive and specific biomarkers for disease monitoring. Circulating cfDNA (circulating free DNA) in plasma contains tumor-derived DNA fragments, holding potential as biomarkers for guiding treatment, monitoring resistance, and early cancer detection (40, 41). ctDNA (circulating tumor DNA) is released into the bloodstream during tumor cell apoptosis or necrosis and is considered a tumor-specific biomarker (42). Both localized and malignant cancers are associated with increased total cfDNA levels, with ctDNA levels typically higher (43, 44). However, some cancer patients may not release detectable ctDNA, and total cfDNA levels can be influenced by factors such as inflammation, liver dysfunction, and kidney disease. These limitations suggest that total cfDNA levels may not reliably serve as standalone biomarkers. As high-risk human papillomavirus (HR-HPV) DNA integrates into the host genome, it is plausible that HPV DNA could be released as ctDNA. Early analyses of HPV DNA in blood showed insufficient sensitivity (45, 46). However, digital PCR (ddPCR) has been increasingly applied in cervical cancer and other HPV-related malignancies (29, 33, 47). Detection techniques, sample sources, and disease staging can significantly impact the accuracy of ctDNA detection, and its prognostic value in cervical cancer remains controversial. For instance, Sivars et al. (28) reported no significant statistical correlation between pre-treatment ctHPV DNA levels and prognosis, while Jeannot E (29) suggested poorer outcomes for cervical cancer patients with detectable pre-treatment ctHPV DNA. Furthermore, Beaussire-Trouvay L (35) found no significant prognostic value of pre-treatment ctDNA detection compared to SCC-A. In contrast, post-treatment ctDNA detection may help identify patients at high risk of recurrence and may correlate with prognosis.

This study systematically reviewed the association between ctDNA and survival outcomes in cervical cancer patients, analyzing 10 articles that included over 700 cases. The meta-analysis results demonstrated a significant correlation between ctDNA fluctuations before and after treatment and patient prognosis. Patients with detectable ctDNA or higher ctDNA levels had poorer overall survival (OS) and progression-free survival/disease-free survival (PFS/DFS), significantly lower than those without detectable ctDNA. Although data on OS were limited to fewer studies, ctDNA detection showed potential in assessing the hazard ratios (HR) for OS and PFS. Furthermore, no significant heterogeneity was found among the included studies, with I² statistics indicating high stability of the results. Influencing factors included study design, tumor stage, timing of ctDNA measurement, and types of prognostic events considered. Despite some variability, the structure of the forest plots strengthened the robustness of the combined results, supporting ctDNA as a reliable independent prognostic biomarker in cervical cancer. Based on these findings, we can conclude that ctDNA can be used preoperatively to assess tumor burden and predict prognosis, with higher ctDNA levels typically associated with poorer survival outcomes. Postoperatively and during treatment, ctDNA can be used to monitor minimal residual disease (MRD), assist in early detection of recurrence risk, and outperform imaging detection. During treatment, dynamic changes in ctDNA can reflect therapeutic response, with persistent increases possibly indicating resistance or disease progression. Additionally, the application of ctDNA in immunotherapy and targeted therapy is expanding, providing precise guidance for personalized treatment.

While this study provides valuable insights into the prognostic role of ctDNA in cervical cancer, several limitations must be acknowledged. First, the small sample size and limited number of studies may constrain the generalizability of these findings. Second, variability in study design, detection methods, and patient characteristics could introduce bias and affect the robustness of the results. Furthermore, inconsistencies in ctDNA measurement timing and threshold definitions complicate result interpretation and comparison. Short follow-up durations in some studies also limit a comprehensive evaluation of the long-term prognostic value of ctDNA. Future research should focus on developing standardized protocols for ctDNA detection, including unified extraction and detection techniques, threshold definitions, and multicenter validation to enhance predictive accuracy and minimize methodological variability. With further advancements, ctDNA holds promise as a more reliable independent prognostic biomarker in clinical practice.

## 5 Conclusions

This meta-analysis confirms the significant association between ctDNA and cervical cancer prognosis, highlighting its value as a specific biomarker. ctDNA demonstrates potential for pre-treatment diagnosis and effective monitoring of therapeutic response and disease progression. Its non-invasive nature supports real-time assessment, and combining it with other biomarkers may enhance accuracy. Large-scale studies are needed to standardize protocols and validate its clinical utility, advancing precision oncology.

## Data Availability

The datasets presented in this study can be found in online repositories. The names of the repository/repositories and accession number(s) can be found in the article/supplementary material.
